# mSphere of Influence: a Controlled Burn—Pathogen Manipulation of the Dynamic Chemistry That Results from Inflammation

**DOI:** 10.1128/mSphere.00110-20

**Published:** 2020-02-26

**Authors:** Neal D. Hammer

**Affiliations:** a Department of Microbiology and Molecular Genetics, Michigan State University, East Lansing, Michigan, USA

**Keywords:** *Salmonella*, anaerobic respiration, gut inflammation, metabolism, pathogens, tetrathionate

## Abstract

Neal Hammer works in the field of bacterial pathogenesis, metabolism, and antibiotic resistance. In this mSphere of Influence article, he reflects on how “Gut inflammation provides a respiratory electron acceptor for *Salmonella*” by Winter and colleagues (S. E. Winter, P. Thiennimitr, M. G. Winter, B. P. Butler, et al., Nature 467:426–429, 2010, https://doi.org/10.1038/nature09415) made an impact on him by demonstrating that Salmonella enterica serotype Typhimurium metabolism is uniquely suited to exploit the chemical by-products that result from the host’s inflammatory response.

## COMMENTARY

I love unusual phenotypes. The phenotypes I find the most fascinating are those that have been exploited for diagnostic purposes. In these cases, a pathogen’s distinct metabolism or physiology provides a way to distinguish it from other microbes, often through some sort of color change or simply growing in a selective medium. In most cases, the mechanisms of a particular diagnostic phenotype are known, but an explanation for it in the context of pathogenesis is not as easily obtained. Such was the case with Salmonella enterica serotype Typhimurium and its ability to utilize tetrathionate as an alternative terminal electron acceptor for anaerobic respiration. *S.* Typhimurium utilization of tetrathionate as an alternative terminal acceptor has been exploited to enrich this pathogen from samples containing mixed populations of microbes since 1923—nearly a century ago. The genes responsible for tetrathionate respiration have been identified, but an explanation for why *S.* Typhimurium utilizes such an unusual terminal electron acceptor for anaerobic respiration was not known. Tetrathionate-dependent respiration is unusual because the molecule is not present in mammals, or so we thought. Previous to this work, researchers believed that tetrathionate respiration supported *S.* Typhimurium proliferation in a rotting carcass or in environments beyond the host. However, “Gut inflammation provides a respiratory electron acceptor for *Salmonella*” demonstrated that tetrathionate was in fact present in the host, though it had to be coaxed into existence via induction of the host’s oxidative inflammatory response (1). The fact that a molecule typically not present in mammals emerges as a by-product of the inflammatory response impacted my thinking about the dynamic chemistry and metabolic potential at the host-pathogen interface. This work serves as powerful reminder that pathogens have evolved elegant systems to exploit whatever advantage becomes available within the host.

A key piece to understanding how tetrathionate could be produced in the host was seemingly in plain sight. Initially, the tetrathionate broth used to enrich for *S.* Typhimurium does not contain tetrathionate. The broth is instead supplemented with thiosulfate, which is present in mammals. In fact, thiosulfate is the product of host detoxification of hydrogen sulfide released by resident, fermenting bacteria in the colon. Upon oxidation, two thiosulfate molecules link together via a disulfide bond, forming tetrathionate. In tetrathionate broth, this is achieved using the strong oxidant iodine. This fact supported the hypothesis that tetrathionate would be present in an oxidized, thiosulfate-containing environment in the host and what better oxidizing agent is there than the respiratory burst of the innate immune system? In a series of elegant experiments that genetically manipulate both the bacterium and host, Winter and colleagues (1) demonstrate that tetrathionate is produced as a result of the innate immune response to *S.* Typhimurium infection. Essentially, the pathogen stimulates the host inflammatory response, and the ensuing oxidation generates an energetically favorable terminal electron acceptor. The appearance of a potent terminal electron acceptor is seized upon by the invading pathogen, which considerably increases its metabolic potential and enhances proliferation, enabling *S.* Typhimurium to outcompete resident colonic bacteria. In total, the work offers a molecular explanation for what was previously considered to be an unusual anaerobic respiratory pathway in *S.* Typhimurium. It also provides a unique perspective into the dynamic chemical composition of the host-pathogen interface.

This work was influential in two ways at different stages in my career. First, it reminded me of the importance of metabolism to pathogenesis. My research focus at the time this work was published were the enzymes Staphylococcus aureus utilizes to aerobically respire, and this paper provided inspiration that what I was studying was not only an interesting topic but an important one. We found that S. aureus also fine-tunes its respiratory pathways to maximize colonization in distinct organs (2). However, we are just beginning to explore the mechanisms that support S. aureus metabolism-dependent, organ-specific colonization. The second major impression was that we must be consistently vigilant of that fact that the host environment changes in response to bacterial infection. In keeping with this, my group has become interested in nutrient sulfur acquisition by S. aureus and is beginning to explore this in other pathogens. A fundamental finding from the work by Winter et al. (1) is that the host inflammatory response will have profound effects on the chemistry of metabolites present at the host-pathogen interface, especially metabolites that are sensitive to redox chemistry. My lab is currently pursuing how the inflammatory response alters abundance of sulfur-containing metabolites in various host organs, and we are cognizant that inflammation may have dramatic effects on the metabolic options available to the pathogen.

Since its publication in 2010, “Gut inflammation provides a respiratory electron acceptor for *Salmonella*” (1) has been cited more than 800 times, underscoring the impactful nature of the work. Andres Bäumler’s laboratory has continued exploring how the metabolism of *S.* Typhimurium is uniquely tailored to outcompete the microbiota in the inflamed gut. Subsequent work demonstrated that ethanolamine is a significant carbon source during infection and that the anaerobic terminal electron acceptor, nitrate, serves as a chemoattractant that orchestrates *S.* Typhimurium motility toward high-energy environments (3, 4). Other high-profile papers extend beyond the realm of *S.* Typhimurium to highlight the importance of anaerobic nitrate respiration in Escherichia coli proliferation in the inflamed gut and revealed that aerobic respiration is important for Citrobacter rodentium colonization in this highly competitive environment (5, 6). Sebastian Winter has established an independent research program that seeks a better understanding of the distinct metabolic networks of resident gut bacteria and pathogens to “edit” or manipulate the gut microbiota. This research has profound potential for the development of novel therapeutic strategies for treating dysbiosis, colitis, and colorectal cancer. All things considered, a tremendous amount of knowledge has been generated from studying a simple, unusual phenotype.
